# Minimum intervention oral care: staging and grading dental carious lesions in clinical practice

**DOI:** 10.1038/s41415-024-7843-4

**Published:** 2024-09-27

**Authors:** Lorraine Emma Molyneux, Avijit Banerjee

**Affiliations:** 41415192448001 Senior Lecturer in Restorative Dentistry, University of Liverpool, School of Dentistry, Institute of Life Course and Medical Sciences, Faculty of Health and Life Sciences, Liverpool University Dental Hospital, Pembroke Place, Liverpool, L3 5PS, UK; 41415192448002 Professor of Cariology and Operative Dentistry, Honorary Consultant/Clinical Lead, Restorative Dentistry, Research Centre of Oral Clinical Translational Sciences/Conservative and MI Dentistry, Faculty of Dentistry, Oral and Craniofacial Sciences, King´s College London, Guy´s Dental Hospital, Great Maze Pond, London, SE1 9RT, UK

## Abstract

Developmental staging of carious lesions is pivotal for appropriate ethical clinical decision-making in contemporary caries management. Accurate assessment of lesion extent/severity (staging) and activity (grading) allows practitioners to provide the most appropriate preventive advice and suitable interventions, enabling the implementation of evidence-based, person-focused, prevention-based, team-delivered and susceptibility-related phased minimum intervention oral care. Minimally invasive dentistry remains an important operative interventive option for cavitated lesions, but intervening at the right stage ensures patients are not started on an irreversible, destructive restorative cycle unnecessarily. This article provides an update on recommended practical methods for staging the extent/severity and grading the activity of dental carious lesions, especially for those clinical teams delivering primary care and needing to navigate remuneration systems.

## Key points

- Summarises the importance of staging (determining the extent/severity) and grading (level of activity) of dental carious lesions.
- Describes practical methods for staging the extent/severity and grading the activity of dental carious lesions.
- Supports clinical decision-making when managing dental caries in primary care settings.

## Dental caries: prevalence

Dental caries is a significant health issue, with over two billion adults and 520 million children world-wide suffering from the untreated condition.^1^ Dental caries remains a significant oral health issue in England, with 27% of individuals over the age of 16 having carious teeth and a prevalence of dental caries in five-year-olds of 29.3%; however, caries experience in some deprived regions was found to be as high as 38.7%.^2^^,^^3^ The associated costs of managing dental caries are significant, with estimates for the global economic burden of caries to be $245 billion, and direct per-person costs for managing dental caries in the UK of $22,910.^4^^,^^5^

## Carious lesion development

Dental caries is a chronic, non-communicable disease resulting from net demineralisation of the dental hard tissues by acidic bacterial products produced during the degradation of low molecular weight carbohydrates.^6^ Bacteria found within the biofilm form a complex ecosystem composed of numerous species of microorganisms.^7^ In health, the biofilm remains in microbial homeostasis with no net demineralisation of the dental hard tissues. Net demineralisation begins to occur when the microbial homeostasis breaks down within an undisrupted biofilm and proliferation of acid-producing organisms occurs in response to local environmental changes favouring these organisms, a state known as dysbiosis.^8^^,^^9^ The first identifiable stage of demineralisation begins with direct dissolution of apatite crystals present on the surface of enamel.^10^ Continued exposure to bacterial acids results in further dissolution of surface apatite crystals, resulting in enlargement of intercrystalline diffusion pathways and preferential demineralisation of subsurface enamel.^11^

Sound enamel, comprised of well-organised hydroxyapatite crystals, creates a relatively optically transparent structure. Following early demineralisation, the enamel structure is altered, becoming more porous, less translucent and exhibiting some surface irregularities.^12^ These changes lead to increased light scattering, resulting in an optical disruption and the appearance of a white spot lesion. Due to the organic components which contribute to the structure of dentine, progression of dentine caries requires proteolysis and damage to the collagenous matrix that is exposed following demineralisation.^13^ Since dentine has smaller and fewer apatite crystals than enamel, and is more porous, the carious lesion progresses faster in dentine than in enamel.^14^

## Diagnosis of dental caries

There can be confusion around the terms diagnosis, detection and assessment. Diagnosis is the result of human, professional summation of all available information.^15^ Current guidance for caries diagnosis is to adopt a multi-step process to help assess the lesion. This involves lesion detection, determining lesion extent/severity and assessment of lesion activity, all combined with establishing the caries risk/susceptibility of the patient.^16^^,^^17^ There are a wide range of protocols and models available, incorporating various caries risk factors and indicators. Most practitioners are familiar with the term ‘risk assessment', which can be carried out at population and patient levels. Risk and susceptibility are often used interchangeably; however, a shift in focus to identify individual pathological and protective ‘susceptibility' factors can better support customised preventive care planning.^18^ A patient-level caries risk/susceptibility assessment should be included as part of the initial patient oral health assessment (Domain 1 of the minimum intervention oral care [MIOC] framework) and then revisited and reviewed at regular intervals as caries susceptibility is not static and there can be significant fluctuations over time.^19^^,^^20^ Adopting a systematic approach to assessing susceptibility of dental disease is advisable. There are a number of indices available to help clinicians to identify individuals at higher susceptibility of active caries.^21^^,^^22^ Clinical judgement is an important part of assessing susceptibility to caries, as even in the presence of a large number of protective factors, certain risk factors, such as reduced salivary flow, recent caries experience and presence of active disease, would place individuals at higher susceptibility of caries development.^16^

Accurate lesion diagnosis enables implementation of best-practice management strategies, where preventive (non-operative and/or micro-invasive) approaches are implemented, and if operative care is indicated, minimally invasive techniques are used.^23^^,^^24^^,^^25^ A concern often reported by dental practitioners about adopting patient-level MIOC and tooth-level minimally invasive operative approaches is around litigation related to the failure of ‘restoring carious lesions adequately'. Although primary care practitioners are adopting more minimally invasive operative techniques, such as selective caries removal and using adhesive bio-interactive restorative materials, there is still a tendency among clinicians to intervene operatively earlier than current evidence and guidance recommends.^26^^,^^27^ It is important to move away from the thinking that placing restorations alone is considered ‘good dentistry' or ‘cures caries'; emphasis must be placed on delivering prevention-based, sustainable care that offers the best long-term outcomes for patients in maintaining their personal oral health.^20^^,^^28^ Clinical negligence cases are brought for a wide range of reasons, including unnecessary or inappropriate treatment, failures or delays in diagnoses, poor communication or record-keeping, as well as not providing indicated professional and preventive care.^29^ With evidence supporting the preventive, person-focused, susceptibility-related, team-delivered MIOC approach,^16^^,^^20^^,^^23^^,^^30^^,^^31^ and an understanding of the undesirable sequelae of unnecessary commencement of the destructive restorative cycle,^28^ implementation of appropriate minimum intervention strategies for the management of dental caries is essential. Identifying, staging and grading carious lesions from the lesion extent, severity and activity information, gleaned from the clinical examination and shared decision-making, alongside the appropriate non-/micro-/minimally invasive caries management approaches, allows practitioners and teams to demonstrate they are following evidence-based best practice to develop appropriate prevention-based, phased, personalised care plans, as well as to support active surveillance of lesions over time.^32^ This falls in line with current periodontal disease management guidelines in primary care.^33^

## Carious lesion detection and severity (staging)

**Fig. 1:**
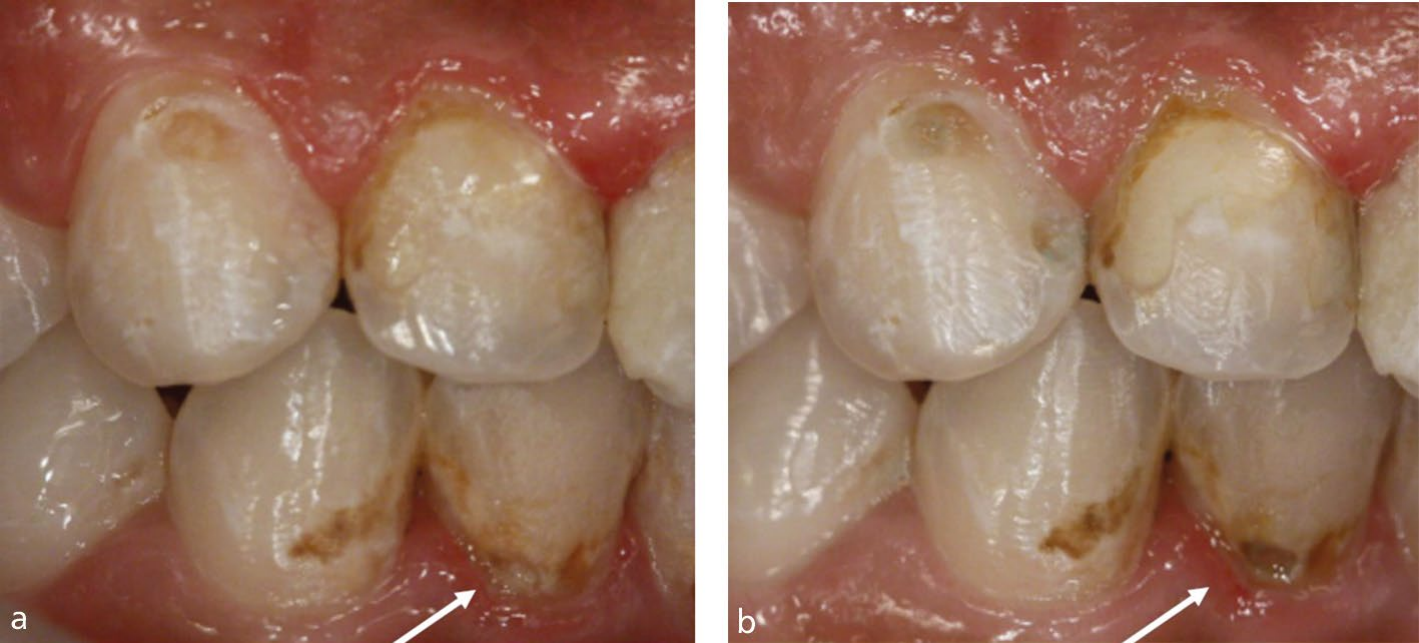
a) Shows teeth on initial presentation. b) Shows the same dentition after plaque removal. The carious lesion on the 42 (arrowed) is more easily detected and the extent can be more accurately determined following plaque removal. The position of the lesion at the gingival margin and biofilm quality and quantity can also inform the carious lesion activity assessment

Visual assessment of carious lesions dates as far back as dentistry itself and is still considered to be the most pragmatic method for clinical lesion detection. For comprehensive caries detection and assessment, teeth should be examined using focused illumination (ideally with magnification), with clean tooth surfaces, requiring the removal of surface debris, such as plaque and calculus. It is important to note the distribution, quality (density, thickness and stickiness) and quantity of biofilm deposits as they are removed, as these help inform caries process activity assessment.^34^^,^^35^ Areas of plaque stagnation/caries-susceptible sites should be examined with particular care; these may vary between individuals but usually include pits and fissures, particularly on partially erupted or submerged teeth, proximal surfaces just cervical to the contact points and smooth surfaces adjacent to the gingival margins (Fig. 1). When examining restored teeth, careful assessment of restoration margins is advised, particularly in the presence of overhangs or deficiencies.

Once the tooth surface is cleaned of any surface debris, in-depth probing of the surface of carious lesions using a sharp dental explorer is not advocated as a diagnostic assessment for carious lesions due to the potential for causing iatrogenic and irreversible cavitation of incipient lesions.^36^ The use of a sharp dental explorer does not improve diagnostic reliability and may affect the potential for lesions to remineralise by the creation of sub-clinical surface defects.^37^^,^^38^ A round-ended probe is advocated instead to check carefully for cavitation by gentle tactile feedback of surface contour and texture, using gentle, lateral sweeping motions over the tooth surface. Teeth should be examined both wet and dry, using the three-in-one air-water syringe, as when the teeth are dried, earlier detection of initial lesions is possible, whereas moderately deep lesions can be easier to identify when the teeth are wet. If there is uncertainty over the presence and extent of lesions on proximal surfaces, temporary tooth separation can be carried out using interproximal wooden wedges or orthodontic separators to separate the teeth to aid visualisation and tactile feedback.^39^^,^^40^

Approaches to caries diagnosis by clinicians have been shown to be variable. A systematic review identified substantial variability in diagnostic criteria and caries concepts worldwide.^41^ To work towards a unified, evidence-based approach towards caries detection, lesion assessment and caries diagnosis, the International Caries Detection and Assessment System (ICDAS) was developed, classifying carious lesions into six distinct categories.^42^ Since its introduction, ICDAS has been widely used across caries research, education and public health.^43^ To further support its implementation into clinical practice, this six-category system was modified into a simpler four-point scoring system (mICDAS)^44^ and the classification system was developed further, incorporating caries management aspects, resulting in the International Caries Classification and Management System^45^ and CariesCare4D, providing a framework for diagnosis and management of carious lesions for dental professionals.^16^ For lesion severity, a staging system classifying lesions as ‘initial', ‘moderate' or ‘extensive' is recommended.^16^^,^^40^ Table 1 summarises the characteristics of coronal and root carious lesion assessment. The relative activity, associated cavitation of a carious lesion and the overall cleansability of the lesion are the three key factors in the diagnostic process and deciding when to intervene. Caries associated with restorations or sealants (CARS; secondary caries) are staged and graded in the same way as coronal primary carious lesions. Where the margins of restorations are unsound, a minimally invasive ‘5Rs' management approach is recommended: reviewing the defect; refurbishment of the surface; and re-sealing, repair or replacement of the tooth-restoration complex.^46^^,^^47^^,^^48^^,^^49^

**Table 1:** Summary of recommended criteria for clinical and radiographic assessment to stage carious lesions and to establish the activity (grade) of carious lesions. Initial carious lesions can be managed non-operatively. Moderate lesions usually require the most consideration regarding whether to adopt non-operative, micro-invasive or minimally invasive operative interventions

**Staging**	**Clinical assessment (with related mICDAS scores)**			
	Sound (mICDAS 0)	Coronal **Figure:** 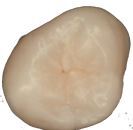	No evidence of caries after air-drying	
		Root **Figure:** 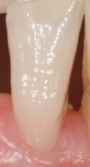		
	Initial (mICDAS 1)	Coronal **Figure:** 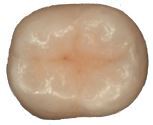	Opacity or discolouration attributable to dental caries after enamel surface air-drying, with no underlying dentine shadowing or cavitation	
		Root **Figure:** 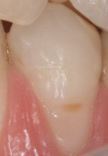	Clear demarcation on root surface with no or minimal change to normal anatomical contour (less than 0.5 mm)	
	Moderate (mICDAS 2, 3)	Coronal **Figure:** 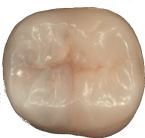	Lesion with shadow of discoloured dentine visible through wet enamel surface or evidence of initial cavitation	
		Root **Figure:** 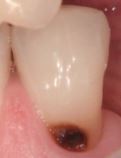	Cavitation present (0.5-2 mm)	
	Extensive (mICDAS 4)	Coronal **Figure:** 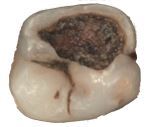	Distinct cavity exposing underlying deeper carious dentine	
		Root **Figure:** 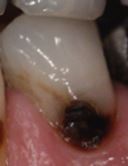	Cavitation present (>2 mm)	
	**Radiographic assessment (with related mICDAS scores)**			
	Sound (mICDAS 0)	**Figure:** 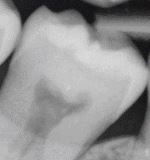	No radiolucency	
	Initial (mICDAS 1,2)	E1 **Figure:** 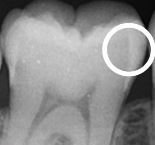	Radiolucency in the outer half of the enamel	
		E2 **Figure:** 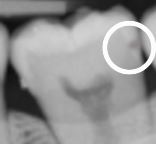	Radiolucency in the inner half of the enamel ± enamel-dentine junction	
		D1 **Figure:** 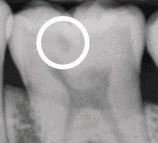	Radiolucency limited to the outer third of the dentine	
	Moderate (mICDAS 3)	D2 **Figure:** 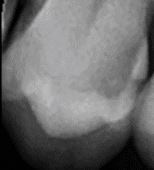	Radiolucency reaching the middle third of the dentine (likely to be cavitated)	
	Extensive (mICDAS 4)	D3 **Figure:** 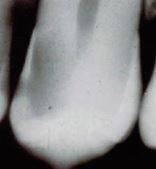	Radiolucency reaching the inner third of the dentine or into the pulp (clinically cavitated)	
**Grading**	**Lesion activity assessment**			
	Activity status		Initial/moderate lesions	Extensive lesions
	Likely inactive/arrested		Enamel surface whitish, brownish or black Enamel shiny Feels hard and smooth to gentle probing across surface Lesion not in a plaque stagnation area	Dentine shiny and hard on gentle probing
	Likely active		Enamel surface whitish or yellowish Opaque appearance Loss of lustre Feels rough to gentle probing across tooth surface Lesion in a plaque stagnation area	Dentine feels soft or leathery on gentle probing

## Radiographic assessment

Radiography is an important supplementary diagnostic aid in detecting carious lesions. The most frequently taken radiographs for lesion detection are bitewings, which are used to screen for proximal surface lesions that are difficult to detect and diagnose clinically. Radiographs also offer an adjunct to clinical examination in determining the severity/depth of lesions, particularly proximal lesions and deeper occlusal lesions, and therefore can assist in decision-making with regards to the most appropriate management strategies.^50^ Determination of lesion progression into dentine is of particular importance; where carious lesions extend to the middle third of the dentine and beyond, cavitation is often (but not always) present and minimally invasive operative interventions are likely to be needed.^51^ For lesions extending up to the outer third of the dentine, which may not be cavitated clinically, the use of preventive or micro-invasive interventions, such as remineralising agents, resin infiltration or therapeutic sealants, should be considered. Table 1 outlines recommended criteria for radiographic extent assessment.^16^^,^^40^

Although visual-tactile clinical assessment for lesion activity is recommended, radiographs can offer some additional diagnostic ‘clues', to be interpreted with care as they will only provide 2D information of a 3D scenario at a given snapshot in time. For example, radiographs can show if tertiary dentine has been laid down in response to ongoing lesion activity. This can be seen by relative shrinkage of the of pulp horns adjacent to the more radiolucent carious demineralisation. A ‘moth-eaten' radiolucent outline at the advancing border of the dentine lesion may also indicate active lesion progression.^44^ If a more defined radio-opaque boundary between the pulp and the lesion boundary can be observed, this could indicate a relative tipping of the metabolic balance towards lesion arrest and mineral deposition. In order to assess lesion behaviour over time, longitudinal radiographs can be used to assess these changes. Care is needed to ensure the alignment of the x-ray beam and the film are duplicated in sequential radiographs - this is not always easy to achieve. Digital subtraction radiography has been suggested as a potential method to improve the active surveillance of lesions over time;^52^^,^^53^ however, further studies are required to assess the validity and reliability of this technique. A disadvantage associated with radiography is exposure of the patient to ionising radiation. The perceived benefits from dental radiography need to be justified against the associated risks; therefore, it is essential that the frequency of screening radiography is tailored to each patient and their individual caries susceptibility.^54^ For active surveillance of carious lesions over time, the depth/extent of the carious lesions under review should also inform the recommended radiographic intervals, with deeper lesions having a shorter interval between radiographs.^35^^,^^55^

## Lesion activity (grading)

The presence of a cariogenic, dysbiotic, dense, stagnant plaque biofilm on a tooth surface is indicative clinically of an overall active caries process. The assessment of carious lesion activity is an important component of the caries diagnostic process as it has significant implications for the optimal management of potentially developing lesions.^56^ Active lesions may be defined as those that exhibit ongoing mineral loss due to metabolic activity in the overlying biofilm and therefore require intervention to rebalance the biofilm dysbiosis and prevent further progression.^51^^,^^57^^,^^58^ In the absence of other indications, such as compromised aesthetics, form and/or function, inactive (arrested) lesions, where there is no net mineral loss occurring or potentially even lesion repair in progress, do not require operative treatment.^50^ To assess lesion activity, there is no single characteristic indicative of an active carious lesion; instead, several clinical factors are considered. These factors include the surface colour, appearance and texture of the lesion as determined by gentle examination of the surface with a round-ended probe, as well as whether it is located in a plaque-stagnation area (Table 1).^16^^,^^35^^,^^40^ Reliable determination of lesion activity at a single visit is challenging as a carious lesion may have features associated with both active and inactive lesions or a lesion could be at a transitional stage between inactive and active.^34^^,^^56^ As part of the activity assessment, clinicians should assess the cleansability of each lesion, as inability to disrupt the biofilm would be indicative of a lesion that is likely to progress.^35^^,^^59^^,^^60^ If any doubt exists regarding the activity status of a lesion, it is recommended to classify the lesion as active and implement appropriate preventive, non-operative management strategies to promote lesion arrest. Due to the dynamic nature of caries, careful longitudinal active surveillance of all carious lesions is recommended to identify and record any changes over time. The most valid method for determining lesion activity is repeated assessment of lesions over time, and this also allows practitioners to determine the success of any non-operative preventive interventions.^35^

## Additional clinical lesion detection and assessment technologies

A range of technologies and methods have been developed to support practitioners in the diagnosis of dental carious lesions. Table 2 outlines some of the existing technologies available for the detection and assessment of carious lesions. While some of these technologies show promise in adjunctive use for the future, careful visual-tactile assessment supplemented with dental radiography where appropriate is still recommended as current base-line assessment in team-delivered MIOC delivery in primary care.^35^ The use of diagnostic tools that allow for the detection of sub-clinical dental carious lesions are not currently recommended in general dental practice as they offer minimal clinical care benefit and could encourage unnecessary interventions.

**Table 2:** Adjunctive technologies available for clinical carious lesion detection and assessment

**Technology**	**Mode of action**	**Advantages**	**Disadvantages**
Fibre-optic transillumination and digital transillumination^15^^,^^61^^,^^62^	Increased light scattering by demineralised dental hard tissues resulting in reduced transmission of light through the tooth	Non-invasive. Allows for immediate interpretation. Colour changes observed may also indicate if lesion extends into dentine	Diagnostic accuracy limited. Some research has shown it to have low sensitivity. Operator experience needed to interpret outcomes
Quantitative light-induced fluorescence^63^^,^^64^^,^^65^	Blue-violet light is directed onto the tooth surface. Causes excitation of fluorophores within dentine resulting in fluorescence of the tooth. Demineralised enamel and dentine exhibit reduced fluorescence. Images are captured and analysed to determine the amount of demineralisation	Non-invasive. Can provide quantitative values informing extent and allowing monitoring over time. Can support early detection of lesions	Some models require a darkened environment. Interpretation can vary between operators. Training needed. Can be affected by staining or restorations
Laser fluorescence^65^^,^^66^^,^^67^^,^^68^	Low energy light is directed at the tooth. Differences observed in energy absorption and subsequent wavelength of emitted light between sound and carious hard tissues	Non-invasive. Immediate feedback. Can provide quantitative values. Intensity of fluorescence detected could indicate lesion extent	Interpretation may vary between operators
Electrical conductance/impedance measurements^68^^,^^69^^,^^70^^,^^71^	A low frequency electrical current is passed through the tooth. Demineralised enamel and dentine contain higher concentrations of fluid/ electrolytes, which are more conductive than sound enamel and dentine. The resulting increase in conductance or reduced impedance of the electrical current through the tooth tissue is measured	Non-invasive. Provides quantitative measurements. Can be used for monitoring carious lesions over time	Sensitivity and specificity shown to be variable
Bioluminescence^72^^,^^73^	Imaging is undertaken to assess light emission resulting from a chemical reaction between photoproteins in solution and free calcium ions released from actively demineralising dental hard tissues. The luminescence is recorded by an intra-oral camera and overlaid onto an image of the tooth surface, providing a map of where active demineralisation is occurring	May provide single-visit assessment of carious lesion activity. Early detection of lesions. Non-invasive. Can allow monitoring over time	Operator training needed
Artificial intelligence^74^^,^^75^^,^^76^	Machine learning algorithms trained on a wide range of digital images analyse images to identify patterns and features associated with carious lesions	Automated and objective. Can analyse large numbers of images quickly. Demonstrated increased sensitivity for enamel lesions; however, this could lead to invasive intervention earlier than indicated	Accuracy dependent upon size and diversity of training dataset. Validation by dental professionals still advised due to risk of false positive or negatives. Limited benefits in detecting moderate and severe carious lesions

## Diagnosis and carious lesion management

As dental caries is a complex and multifactorial biofilm-mediated disease, a significant challenge for clinicians is to ascertain the relative importance of each of the contributory factors involved in a particular patient and subsequently to determine the most effective strategies for diagnosis, prevention and treatment at the individual patient level.^77^ Once diagnoses have been made, practitioners should follow best practice guidance in determining the prognosis and optimal management strategies for the patient and for any identified lesions.^16^^,^^51^ Activity, whether the lesion is cavitated or not, and its cleansability, are the three most significant factors in determining what interventions are recommended.^50^ Preventive methods using the MIOC approach should aim to address as many of the aetiological factors as possible, including supporting patient behaviour change with goal-setting, disrupting or modulating the biofilm, modifying diet and promoting remineralisation.^32^^,^^78^ Active surveillance alone may be indicated where lesions are determined to be inactive, as further mineral loss is unlikely.^79^ In the absence of symptoms or other patient complaints, even frank cavitation does not necessarily indicate operative intervention; if lesions are cleansable and an adequate standard of oral hygiene can be achieved, lesions may be able to arrest and remineralise with non-operative and micro-invasive (non-restorative cavity control) interventions.^51^^,^^80^^,^^81^^,^^82^^,^^83^^,^^84^

## Summary

The role of oral healthcare team members in caries management is becoming increasingly focused on preventing caries developing in highly susceptible individuals and managing early lesions using non-invasive or micro-invasive methods to remove the ultimate need for operative intervention.^85^ With oral and dental healthcare delivery centred on prevention-based, susceptibility-related, person-focused, team-delivered MIOC, early detection and detailed assessment of lesions is essential.^32^ Table 1 summarises the recommended criteria to support practitioners in assessing carious lesion extent/severity (staging) and its activity (grading). Decision-making regarding appropriate interventions for all carious lesions should follow an assessment of lesion staging, grading, level of cavitation and related cleansability, alongside determination of the caries susceptibility status of the individual.^32^^,^^51^^,^^57^ This best clinical practice will then allow the correct allocation of resources in primary care settings.

## References

[1] Global Burden of Disease Collaborative Network. *Global Burden of Disease Study 2019 (GBD 2019).* Seattle: Institute for Health Metrics and Evaluation, 2020.

[2] UK Government. Oral health survey of adults attending general dental practices 2018. 2020. Available at https://www.gov.uk/government/publications/oral-health-survey-of-adults-attending-dental-practices-2018 (accessed August 2024).

[3] UK Government. National Dental Epidemiology Programme (NDEP) for England: oral health survey of 5-year-old children 2022. 2023. Available at https://www.gov.uk/government/statistics/oral-health-survey-of-5-year-old-children-2022/national-dental-epidemiology-programme-ndep-for-england-oral-health-survey-of-5-year-old-children-2022 (accessed August 2024).

[4] Alliance for a Cavity-Free Future. An economic perspective of the global burden of dental caries. 2021. Available at https://www.acffglobal.org/wp-content/uploads/2021/03/An-economic-perspective-on-the-burden-of-dental-caries.pdf (accessed August 2024).

[5] European Federation of Periodontology. Time to put your money where your mouth is: addressing inequalities in oral health. 2024. Available at https://impact.economist.com/perspectives/sites/default/files/eixefp_-_time_to_put_money_where_mouth_is_addressing_inequalities_in_oral_health_white_paper_v2.pdf (accessed August 2024).

[6] Ten Cate J M, Larsen M J, Pearce E I F, Fejerskov O. Chemical interactions between the tooth and oral fluids. *In* Fejerskov O, Kidd E (eds) *Dental Caries: The Disease and Its Clinical**Management*. 2nd ed. pp 209-231. Oxford: Blackwell-Munksgaard, 2008.

[7] Marsh P D. Dental plaque: biological significance of a biofilm and community life-style. *J Clin Periodontol* 2005; **32:** 7-15. 10.1111/j.1600-051X.2005.00790.x 16128825

[8] Takahashi N, Nyvad B. The role of bacteria in the caries process: ecological perspectives. *J Dent Res* 2011; **90:** 294-303. 10.1177/0022034510379602 20924061

[9] Marsh P D, Zaura E. Dental biofilm: ecological interactions in health and disease. *J Clin Periodontol* 2017; **44:** 12-22. 10.1111/jcpe.12679 28266111

[10] Thylstrup A, Bruun C, Holmen L. *In vivo* caries models-mechanisms for caries initiation and arrestment. *Adv Dent Res* 1994; **8:** 144-157. 10.1177/08959374940080020401 7865069

[11] Mann A B, Dickinson M E. Nanomechanics, chemistry and structure at the enamel surface. *In* Duckworth R M (ed) *The Teeth and Their Environment*. *Monographs in Oral Science.* Vol 19. pp 105-131. Basel: Karger, 2006. 10.1159/000090588 16374031

[12] Fejerskov O. Pathology of dental caries. *In* Fejerskov O, Nyvad B, Kidd E (eds) *Dental Caries: The Disease and Its Clinical Management.* 3rd ed. pp 49-81. Oxford: Wiley-Blackwell, 2015.

[13] Fejerskov O, Larsen M J. Demineralization and remineralization: the key to understanding clinical manifestations of dental caries. *In* Fejerskov O, Nyvad B, Kidd E (eds) *Dental Caries: The Disease and Its Clinical Management.* 3rd ed. pp 155-170. Oxford: Wiley-Blackwell, 2015.

[14] Øgaard B, Rølla G, Arends J. *In vivo* progress of enamel and root surface lesions under plaque as a function of time. *Caries Res* 1988; **22:** 302-305. 10.1159/000261125 3180162

[15] Gomez J. Detection and diagnosis of the early caries lesion. *BMC Oral Health* 2015; **15:** 3. 10.1186/1472-6831-15-S1-S3 PMC4580848 26392124

[16] Martignon S, Pitts N B, Goffin G *et al*. CariesCare practice guide: consensus on evidence into practice. *Br Dent J* 2019; **227:** 353-362. 10.1038/s41415-019-0678-8 31520031

[17] Pitts N, Melo P, Martignon S, Ekstrand K, Ismail A. Caries risk assessment, diagnosis and synthesis in the context of a European Core Curriculum in Cariology. *Eur J Dent Educ* 2011; **15:** 23-31. 10.1111/j.1600-0579.2011.00711.x 22023543

[18] Doméjean S, Banerjee A, Featherstone J D B. Caries risk/susceptibility assessment: its value in minimum intervention oral healthcare. *Br Dent J* 2017; **223:** 191-197. 10.1038/sj.bdj.2017.665 28798458

[19] Twetman S, Fontana M, Featherstone J D B. Risk assessment - can we achieve consensus? *Community Dent Oral Epidemiol* 2013; **41:** 64-70. 10.1111/cdoe.12026 24916679

[20] Leal S, Damé-Teixeira N, Barbosa C *et al*. Minimum intervention oral care: defining the future of caries management. *Braz Oral Res* 2022; **36:** 135. 10.1590/1807-3107bor-2022.vol36.0135 36383841

[21] Su N, Lagerweij M D, van der Heijden G J M G. Assessment of predictive performance of caries risk assessment models based on a systematic review and meta-analysis. *J Dent* 2021; **110:** 103664. 10.1016/j.jdent.2021.103664 33984413

[22] Twetman S, Banerjee A. Caries Risk Assessment. *In* Chapple I L, Papapanou P N (eds) *Risk Assessment in Oral Health: A Concise Guide for Clinical Application*. Cham: Springer, 2020.

[23] Banerjee A, Frencken J E, Schwendicke F, Innes N P T. Contemporary operative caries management: consensus recommendations on minimally invasive caries removal. *Br Dent J* 2017; **223:** 215-222. 10.1038/sj.bdj.2017.672 28798430

[24] Innes N P T, Schwendicke F. Restorative thresholds for carious lesions: systematic review and meta-analysis. *J Dent Res* 2017; **96:** 501-508. 10.1177/0022034517693605 28195749

[25] Banerjee A. ‘MI'opia or 20/20 vision? *Br Dent J* 2013; **214:** 101-105. 10.1038/sj.bdj.2013.105 23392021

[26] Laske M, Opdam N, Bronkhorst E *et al*. Minimally invasive intervention for primary caries lesions: are dentists implementing this concept? *Caries Res* 2019; **53:** 204-216. 10.1159/000490626 PMC6425814 30107377

[27] Chana P, Orlans M C, O'Toole S, Domejean S, Movahedi S, Banerjee A. Restorative intervention thresholds and treatment decisions of general dental practitioners in London. *Br Dent J* 2019; **227:** 727-732. 10.1038/s41415-019-0849-7 31654011

[28] Elderton R J. Preventive (evidence-based) approach to quality general dental care. *Med Princ Pract* 2003; **12:** 12-21. 10.1159/000069841 12707497

[29] Blanchard J R, Koshal S, Navaratnam A, Machin J T, Briggs T W R, Jones E. Hospital dentistry litigation in England: clinical negligence claims against the NHS 2015-2020. *Br Dent J* 2022; DOI: 10.1038/s41415-022-4965-4. 10.1038/s41415-022-4965-4 36068267

[30] Banerjee A. Minimum Intervention (MI) Oral Healthcare Delivery Implementation - Overcoming the Hurdles. *Prim Dent J* 2017; **6:** 28-33. 10.1308/205016817821930944 28987150

[31] Banerjee A. Minimum intervention oral healthcare delivery - is there consensus? *Br Dent J* 2020; **229:** 393-395. 10.1038/s41415-020-2235-x PMC7546164 33037331

[32] Banerjee A, Hameed Z, Chohan M A *et al*. Minimum intervention oral care - incentivising preventive management of high-needs/high caries-risk patients using phased courses of treatment. *Br Dent J* 2024; **236:** 379-382. 10.1038/s41415-024-7132-2 PMC10923692 38459308

[33] West N, Chapple I, Claydon N *et al*. BSP implementation of European S3-level evidence-based treatment guidelines for stage I-III periodontitis in UK clinical practice. *J Dent* 2021; **106:** 103562. 10.1016/j.jdent.2020.103562 33573801

[34] Nyvad B, Baelum V. Nyvad Criteria for Caries Lesion Activity and Severity Assessment: A Validated Approach for Clinical Management and Research. *Caries Res* 2018; **52:** 397-405. 10.1159/000480522 29506010

[35] Neuhaus K W, Kühnisch J, Banerjee A *et al*. Organization for Caries Research-European Federation of Conservative Dentistry Consensus Report on Clinical Recommendations for Caries Diagnosis Paper II: Caries Lesion Activity and Progression Assessment. *Caries Res* 2024; DOI: 10.1159/000538619. 10.1159/000538619 PMC11446318 38684147

[36] Yassin O M. *In vitro* studies of the effect of a dental explorer on the formation of an artificial carious lesion. *ASDC J Dent Child* 1995; **62:** 111-117. 7608368

[37] Lussi A. Comparison of different methods for the diagnosis of fissure caries without cavitation. *Caries Res* 1993; **27:** 409-416. 10.1159/000261572 8242679

[38] Warren J J, Levy S M, Wefel J S. Explorer probing of root caries lesions: an *in vitro* study. *Spec Care Dentist* 2003; **23:** 18-21. 10.1111/j.1754-4505.2003.tb00284.x 12887149

[39] Hintze H, Wenzel A, Danielsen B, Nyvad B. Reliability of visual examination, fibre-optic transillumination, and bite-wing radiography, and reproducibility of direct visual examination following tooth separation for the identification of cavitated carious lesions in contacting approximal surfaces. *Caries Res* 1998; **32:** 204-209. 10.1159/000016454 9577986

[40] Banerjee A. *A Clinical Guide to Advanced Minimum Intervention Restorative Dentistry*. Amsterdam: Elsevier, 2024. 10.1038/s41415-024-8183-0 39672849

[41] Ismail A I. Visual and visuo-tactile detection of dental caries. *J Dent Res* 2004; DOI: 10.1177/154405910408301s12. 10.1177/154405910408301s12 15286124

[42] Ismail A I, Sohn W, Tellez M *et al*. The International Caries Detection and Assessment System (ICDAS): an integrated system for measuring dental caries. *Community Dent Oral Epidemiol* 2007; **35:** 170-178. 10.1111/j.1600-0528.2007.00347.x 17518963

[43] Pitts N B, Banerjee A, Mazevet M E, Goffin G, Martignon S. From ‘ICDAS' to ‘CariesCare International': the 20-year journey building international consensus to take caries evidence into clinical practice. *Br Dent J* 2021; **231:** 769-774. 10.1038/s41415-021-3732-2 PMC8680063 34921275

[44] Banerjee A, Watson T. *Pickard's Guide to Minimally Invasive Operative Dentistry*. 10th ed. Oxford: Oxford University Press, 2015.

[45] Ismail A I, Pitts N B, Tellez M *et al*. The International Caries Classification and Management System (ICCMS) An Example of a Caries Management Pathway. *BMC Oral Health* 2015; **15:** 9. 10.1186/1472-6831-15-S1-S9 PMC4580843 26391116

[46] Banerjee A. *Chapter 9 MIOC Domain 4: Clinical Re-assessment, Recall and Maintenance of the Tooth-Restoration Complex**. In**Banerjee A**(ed) A Clinical Guide to Advanced Minimum Intervention Restorative Dentistry*. pp 253-273. Amsterdam: Elsevier, 2024.

[47] Green D, Mackenzie L, Banerjee A. Minimally Invasive Long-Term Management of Direct Restorations: the ‘5 Rs'. *Dent Update* 2015; **42:** 413-426. 10.12968/denu.2015.42.5.413 26964443

[48] Martins B M C, Silva E J N L D, Ferreira D M T P, Reis K R, Fidalgo T K D S. Longevity of defective direct restorations treated by minimally invasive techniques or complete replacement in permanent teeth: a systematic review. *J Dent* 2018; **78:** 22-30. 10.1016/j.jdent.2018.09.001 30189230

[49] Askar H, Krois J, Göstemeyer G *et al*. Secondary caries: what is it, and how it can be controlled, detected, and managed? *Clin Oral Investig* 2020; **24:** 1869-1876. 10.1007/s00784-020-03268-7 32300980

[50] Schwendicke F, Splieth C, Breschi L *et al*. When to intervene in the caries process? An expert Delphi consensus statement. *Clin Oral Investig* 2019; **23:** 3691-3703. 10.1007/s00784-019-03058-w 31444695

[51] Banerjee A, Splieth C, Breschi L *et al*. When to intervene in the caries process? A Delphi consensus statement. *Br Dent J* 2020; **229:** 474-482. 10.1038/s41415-020-2220-4 33037372

[52] Neuhaus K W, Longbottom C, Ellwood R, Lussi A. Novel lesion detection aids. *In* Pitts N B (eds) *The Teeth and Their Environment*. *Monographs in Oral Science**. Vol 21.* pp 52-62. Basel: Karger, 2009. 10.1159/000224212 19494675

[53] Carneiro L S, Nunes C A, Silva M, Leles C R, Mendonça E F. *In vivo* study of pixel grey-measurement in digital subtraction radiography for monitoring caries remineralization. *Dentomaxillofac Radiol* 2009; **38:** 73-78. 10.1259/dmfr/15857365 19176648

[54] Faculty of General Dental Practice. *Selection Criteria for Dental Radiography*. London: FGDP, 2018.

[55] Kühnisch J, Anttonen V, Duggal M S *et al*. Best clinical practice guidance for prescribing dental radiographs in children and adolescents: an EAPD policy document. *Eur Arch Paediatr Dent* 2020; **21:** 375-386. 10.1007/s40368-019-00493-x 31768893

[56] Drancourt N, Roger-Leroi V, Martignon S, Jablonski-Momeni A, Pitts N, Doméjean S. Carious lesion activity assessment in clinical practice: a systematic review. *Clin Oral Investig* 2019; **23:** 1513-1524. 10.1007/s00784-019-02839-7 30790086

[57] Nyvad B, Machiulskiene V M, Soviero V M, Baelum V. Visual-tactile caries diagnosis. *In* Fejerskov O, Nyvad B, Kidd E (eds) *Dental Caries: The Disease and Its Clinical Management.* 3rd ed. pp 191-210. Oxford: Wiley-Blackwell, 2015.

[58] Machiulskiene V, Campus G, Carvalho J C *et al*. Terminology of Dental Caries and Dental Caries Management: Consensus Report of a Workshop Organized by ORCA and Cariology Research Group of IADR. *Caries Res* 2020; **54:** 7-14. 10.1159/000503309 31590168

[59] Nyvad B, Machiulskiene V, Baelum V. Reliability of a new caries diagnostic system differentiating between active and inactive caries lesions. *Caries Res* 1999; **33:** 252-260. 10.1159/000016526 10343087

[60] Ekstrand K R, Martignon S, Ricketts D J, Qvist V. Detection and activity assessment of primary coronal caries lesions: a methodologic study. *Oper Dent* 2007; **32:** 225-235. 10.2341/06-63 17555173

[61] Verdonschot E H, Bronkhorst E M, Wenzel A. Approximal caries diagnosis using fiber-optic transillumination: a mathematical adjustment to improve validity. *Community Dent Oral Epidemiol* 1991; **19:** 329-332. 10.1111/j.1600-0528.1991.tb00181.x 1837257

[62] Vaarkamp J, ten Bosch J J, Verdonschot E H, Bronkhorst E M. The real performance of bitewing radiography and fiber-optic transillumination in approximal caries diagnosis. *J Dent Res* 2000; **79:** 1747-1751. 10.1177/00220345000790100301 11077989

[63] Pretty I A. Caries detection and diagnosis: novel technologies. *J Dent* 2006; **34:** 727-739. 10.1016/j.jdent.2006.06.001 16901606

[64] De Josselin de Jong E, Sundström F, Westerling H, Tranæus S, ten Bosch J J, Angmar-Månsson B. A new method for in vivo quantification of changes in initial enamel caries with laser fluorescence. *Caries Res* 1995; **29:** 2-7. 10.1159/000262032 7867045

[65] Shi X Q, Tranæus S, Angmar-Månsson B. Comparison of QLF and DIAGNOdent for Quantification of Smooth Surface Caries. *Caries Research* 2001; **35:** 21-26. 10.1159/000047426 11125192

[66] Lussi A, Megert B, Longbottom C, Reich E, Francescut P. Clinical performance of a laser fluorescence device for detection of occlusal caries lesions. *Eur J Oral Sci* 2001; **109:** 14-9. 10.1034/j.1600-0722.2001.109001014.x 11330928

[67] Hibst R, Paulus R, Lussi A. Detection of occlusal caries by laser fluorescence: Basic and clinical investigations. *Med Laser Appl* 2001; **16:** 205-213.

[68] Hintze H, Lussi A, Cuisinier F, Nyvad B. Additional caries detection methods*. In* Fejerskov O, Nyvad B, Kidd E (eds) *Dental Caries: The Disease and Its Clinical Management*. 3rd ed. pp 211-234. Oxford: Wiley-Blackwell, 2015.

[69] Macey R, Walsh T, Riley P *et al*. Electrical conductance for the detection of dental caries. *Cochrane Database Syst Rev* 2021; **3:** Cd014547. 10.1002/14651858.CD014547 PMC8406820 33724442

[70] Longbottom C, Huysmans M C. Electrical measurements for use in caries clinical trials. *J Dent Res* 2004; **83:** 76-79. 10.1177/154405910408301s15 15286127

[71] Kockanat A, Unal M. In vivo and in vitro comparison of ICDAS II, DIAGNOdent pen, CarieScan PRO and SoproLife camera for occlusal caries detection in primary molar teeth. *Eur J Paediatr Dent* 2017; **18:** 99-104. 10.23804/ejpd.2017.18.02.03 28598179

[72] Pitts N, Shanks N, Longbottom C, Willins M, Vernon B. Clinical validation of a novel bioluminescence imaging technology for aiding the assessment of carious lesion activity status. *Clin Exp Dent Res* 2021; 7: 772-785. 10.1002/cre2.400 PMC8543484 33689205

[73] Longbottom C, Vernon B. Chapter 22: Bioluminescence technology to aid lesion activity assessment. *In* Zandona A F, Longbottom C (eds) *Detection and Assessment of Dental Caries: A Clinical Guide.* pp 217-224. Cham: Springer, 2019.

[74] Khanagar S B, Al-ehaideb A, Maganur P C *et al*. Developments, application, and performance of artificial intelligence in dentistry - a systematic review. *J Dent Res* 2021; **16:** 508-522. 10.1016/j.jds.2020.06.019 PMC7770297 33384840

[75] Schwendicke F, Samek W, Krois J. Artificial Intelligence in Dentistry: Chances and Challenges. *J Dent Res* 2020; **99:** 769-774. 10.1177/0022034520915714 PMC7309354 32315260

[76] Mertens S, Krois J, Cantu A G, Arsiwala LT, Schwendicke F. Artificial intelligence for caries detection: Randomized trial. *J Dent*. 2021; **115:** 103849. 10.1016/j.jdent.2021.103849 34656656

[77] Kiberstis P, Roberts L. It's not just the genes. *Science* 2002; **296:** 685.

[78] Paris S, Meyer-Lueckel H. Paradigm shift in cariology. *In* Meyer-Lueckel H, Paris S, Ekstrand K (eds) *Caries Management - Science and Clinical Practice*. pp 65-68. Stuttgart: Thieme, 2013.

[79] Nyvad B, Kidd E A M. The principles of caries control for the individual patient. *In* Fejerskov O, Nyvad B, Kidd E (eds) *Dental Caries: The Disease and Its Clinical Management*. 3rd ed. pp 303-320. Oxford: Wiley-Blackwell, 2015.

[80] Nyvad B, Fejerskov O. Active root surface caries converted into inactive caries as a response to oral hygiene. *Eur J Oral Sci* 1986; **94:** 281-284. 10.1111/j.1600-0722.1986.tb01765.x 3461550

[81] Meyer-Lueckel H, Machiulskiene V, Giacaman R A. How to Intervene in the Root Caries Process? Systematic Review and Meta-Analyses. *Caries Res* 2019; **53:** 599-608. 10.1159/000501588 PMC6943811 31412343

[82] Splieth C H, Banerjee A, Bottenberg P *et al*. How to Intervene in the Caries Process in Children: A Joint ORCA and EFCD Expert Delphi Consensus Statement. *Caries Res* 2020; **54:** 297-305. 10.1159/000507692 32610317

[83] Paris S, Banerjee A, Bottenberg P *et al*. How to Intervene in the Caries Process in Older Adults: A Joint ORCA and EFCD Expert Delphi Consensus Statement. *Caries Res* 2020; **54:** 1-7. 10.1159/000510843 33291110

[84] Schwendicke F, Splieth C H, Bottenberg P *et al*. How to intervene in the caries process in adults: proximal and secondary caries? An EFCD-ORCA-DGZ expert Delphi consensus statement. *Clin Oral Investig* 2020; **24:** 3315-3321. 10.1007/s00784-020-03431-0 32643090

[85] Ricketts D N J, Pitts N B. Traditional operative treatment options. *In* Pitts N (ed) *The Teeth and Their Environment*. *Monographs in Oral Science*. Vol 21. pp 164-173. Basel: Karger, 2009. 10.1159/000224221 19494684

